# Sodium-Dependent Conformational Change in Flagellar Stator Protein MotS from *Bacillus subtilis*

**DOI:** 10.3390/biom15020302

**Published:** 2025-02-18

**Authors:** Norihiro Takekawa, Ayaka Yamaguchi, Koki Nishiuchi, Maria Uehori, Miki Kinoshita, Tohru Minamino, Katsumi Imada

**Affiliations:** 1 Department of Macromolecular Science, Graduate School of Science, Osaka University, 1-1 Machikaneyama, Toyonaka 560-0043, Osaka, Japan; takekawan16@chem.sci.osaka-u.ac.jp (N.T.);; 2 Graduate School of Frontier Biosciences, Osaka University, 1-3 Yamadaoka, Suita 565-0871, Osaka, Japan; kinoshita.miki.fbs@osaka-u.ac.jp (M.K.); tohru@fbs.osaka-u.ac.jp (T.M.)

**Keywords:** stator, gram-positive bacteria, flagellum

## Abstract

The bacterial flagellar motor consists of a rotor and stator units and is driven by ion flow through the stator. The activation of the ion flow is coupled with the anchoring of the stator units to the peptidoglycan layer by the stator B-subunit around the rotor. Gram-negative bacteria, such as *Salmonella* and *Vibrio*, change the conformation of the N-terminal helix of the periplasmic domain of the B-subunit to anchor the stator units. However, a recent high-speed atomic force microscopic study has suggested that the periplasmic domain of MotS, the stator B-subunit of the sodium (Na^+^)-driven stator of *Bacillus subtilis*, a gram-positive bacterium, unfolds at low external Na^+^ concentrations and folds at high Na^+^ concentrations to anchor the stator units. Here, we report the crystal structures of MotS_68–242_, a periplasmic fragment of MotS, from *B. subtilis* at high and low Na^+^ concentrations. We also performed far-UV CD spectroscopic analysis of the wild-type MotS_68–242_ and MotS_78–242_ proteins and mutant variants of MotS_68–242_ under high and low Na^+^ concentrations and found that the N-terminal disordered region of MotS_68–242_ shows a Na^+^-dependent coil–helix transition. We propose a mechanism of the Na^+^-dependent structural transition of Bs-MotS to anchor the stator units.

## 1. Introduction

Flagellar motility is important for virulence, competitiveness, and survival for many bacteria. The flagellum consists of a helical filamentous axial structure extending from the cell body and a reversible motor embedded in the cell membrane at the base of the axial structure [1,2]. The axial structure is rotated by the motor powered by electrochemical potential energy across the cell membrane. The motor is composed of a rotor and a dozen stator units surrounding the rotor [3]. The rotor comprises the MS-ring in the cytoplasmic membrane and the C-ring attached beneath the MS-ring. The MS-ring consists of a transmembrane protein, FliF, and acts as an assembly base for the flagellum. The C-ring is made of three cytoplasmic proteins, FliG, FliM, and FliN, and is essential for torque generation by interacting with the stator units. The stator unit is an ion channel that converts the electrochemical potential of a specific ion, such as proton (H^+^) or sodium ions (Na^+^), into mechanical rotation. The stator unit consists of two types of membrane proteins (the A- and B-subunits) [4] called MotA and MotB for the H^+^-driven stator [5], PomA and PomB for the Na^+^-driven stator from *Vibrio* species [6], and MotP and MotS for the Na^+^-driven stator from some *Bacillus* species [7].

**Figure 1:**
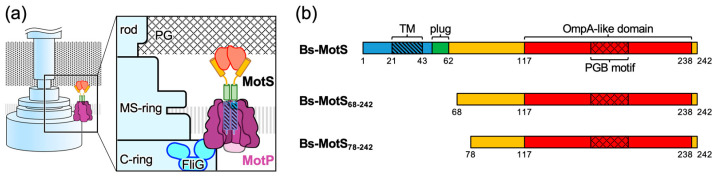
Flagellar motor and the stator protein MotS from *B. subtilis*. (**a**) Schematic diagram of the flagellar motor of *B. subtilis*. The motor consists of the rotor (C-ring, MS-ring, and rod) and the stator (MotP and MotS). (**b**) Schematic representation of the primary structure of *B. subtilis* MotS (Bs-MotS) and its fragments used in this study. TM, transmembrane region; PGB motif, peptidoglycan-binding motif.

CryoEM structural studies of the stator unit revealed that the stator unit is a hetero-heptameric complex in which the transmembrane helices of the B-subunit dimer penetrate the central hole of the pentameric A-subunit ring [8,9,10,11]. The rotational symmetry mismatch between the A- and B-subunits has led to a model of the flagellar rotation mechanism in which rotation of the A-subunit ring around the B-subunit dimer coupled with specific ion influx drives the rotor rotation by the interaction between the stator A-subunit and rotor protein FliG in the C-ring [8,9,10,11,12]. The A-subunit is made up of four transmembrane helices and a cytoplasmic domain [13], which has conserved charged residues essential for interactions with FliG for torque generation [14,15,16,17]. The B-subunit consists of an N-terminal transmembrane helix that contains an aspartate residue critical for specific ion flux through the stator [6,18,19] and a C-terminal periplasmic region containing an OmpA-like domain that binds to the peptidoglycan (PG) layer through the peptidoglycan binding (PGB) motif and/or the T-ring to anchor the stator unit around the rotor [20,21,22,23] (Figure 1). The transmembrane helix of the B-subunit forms an ion pathway with the transmembrane region of the A-subunits [11,18,24,25] An amphiphilic helix following the transmembrane helix of the B-subunit, namely, the plug, blocks the ion flow before incorporation of the stator into the motor [26,27,28,29].

The stator units dynamically associate with and dissociate from the functioning motor, whereas they are anchored to the PG layer or the T-ring for torque generation [30,31,32]. The ion flow through the stator unit is activated only when the stator unit is installed into the motor [25]. Structural studies of a periplasmic fragment of *Salmonella* MotB (St-MotBc), which covers the essential periplasmic region for motor function, and a corresponding fragment of *Vibrio alginolyticus* PomB (Va-PomBc) revealed that they consist of a single core domain with N-terminal long and short α-helices and form a compact dimer [20,21,33]. Because the dimer is too short to reach the PG layer, a conformational rearrangement is expected in the N-terminal helix of the periplasmic region of the B-subunit. An in vivo crosslinking study of Va-PomB indicated that the N-terminal helix adopts a conformational change upon stator incorporation into the motor [20]. Crystallographic and NMR studies of the periplasmic fragment of the St-MotB mutant protein and in vivo crosslinking analysis revealed that the coil–helix transition of the N-terminal helix of St-MotB_C_ occurs to anchor the stator [21].

*Bacillus subtilis* is a gram-positive bacterium and has two types of stator units: H^+^-type MotAB (Bs-MotAB) and Na^+^-type MotPS (Bs-MotPS) [7,34]. The motor driven by Bs-MotAB is dominant under normal swimming conditions, whereas that driven by Bs-MotPS is observed under high pH, high external Na^+^ concentration, and highly viscous conditions [7,34,35]. Bs-MotPS assembles around the rotor depending on the external Na^+^ concentration [34]. Bs-MotS consists of 242 amino acids. Residues 21 to 43 form a transmembrane region, and residues 44 to the C-terminus form a periplasmic region. Residues 117 to 238 show sequence similarity to the OmpA-like domain containing the conserved PGB motif (Figure 1 and Figure S1). Recently, based on direct observations of purified Bs-MotPS by high-speed atomic force microscopy (HS-AFM), a Na^+^-dependent structural change in the periplasmic region of Bs-MotS (Bs-MotSc) has been proposed [36]. In the model, Bs-MotSc is unfolded at low Na^+^ concentrations, but at more than 100 mM NaCl, it folds and forms a dimer that can be anchored to the PG layer. However, the mechanism of the structural change remains unclear, and it is still unclear whether Na^+^-dependent folding really occurs. Moreover, no high-resolution structure of the periplasmic region of the stator B-subunit of gram-positive bacteria has been reported, although the PG layer of gram-positive bacteria differs considerably from that of gram-negative bacteria in its thickness, structure, and composition.

In this study, we determined the structure of MotS_68–242_, a periplasmic fragment of MotS, at high and low Na^+^ concentrations and investigated the mechanism of the Na^+^-dependent structural change using far-UV CD spectroscopy. Our results indicate that the sodium-dependent coil–helix transition occurs in the region connecting the plug and the N-terminal helix of Bs-MotSc. Based on the results, we propose a new model for the sodium-dependent structural change in Bs-MotS that anchors the stator.

## 2. Materials and Methods

### 2.1. Bacterial Strains, Plasmids, and Mutagenesis

The bacterial strains and plasmids used in this study are listed in Table S1. The expression plasmids of *B. subtilis* MotS_68–242_ and MotS_78–242_ (in which the DNA encoding MotS_68–242_ or MotS_78–242_ was inserted between the *Nde*I restriction site and 6 × His coding region of the pET-21b vector) were kindly gifted by Dr. Naoya Terahara of Chuo University. Point mutations in the plasmids were introduced by QuikChange site-directed mutagenesis (Agilent Technologies, Santa Clara, CA, USA). The transformation of *E. coli* was performed using a standard heat shock method.

### 2.2. Protein Expression and Purification

BL21(DE3) cells carrying the expression plasmids of MotS_68–242_, MotS_68–242_(D70A), MotS_68–242_(E75A), MotS_68–242_(D70A/E75A), or MotS_78–242_ were cultured in LB broth containing 50 μg mL^−1^ ampicillin at 37 °C to an optical density of 0.6–0.8 at 660 nm. IPTG was subsequently added to the culture (final concentration: 0.3 mM), and culturing was continued for about 3 h at 30 °C. Cells were harvested by centrifugation (6000× *g* for 10 min) and suspended in buffer A [20 mM Tris-HCl (pH 8.0)] containing 5 mM imidazole and 150 mM NaCl. The cells were then disrupted by sonication on ice. After the removal of cell debris by centrifugation, the cell lysate was loaded onto a HisTrap column (Cytiva, Tokyo, Japan) equilibrated with buffer A containing 5 mM imidazole and 150 mM NaCl, and bound proteins were eluted by a linear 5–500 mM gradient of imidazole in buffer A with 150 mM NaCl. Peak fractions were pooled and concentrated by ultrafiltration using an Amicon Ultra-15 device (Merck Millipore, Tokyo, Japan). The protein sample was further purified with size exclusion chromatography using a Superdex 75 10/300 GL column (Cytiva, Tokyo, Japan) in buffer A with 150 mM NaCl, 300 mM NaCl, or 300 mM KCl. The peak fractions were collected and concentrated to 20 mg mL^−1^ using an Amicon Ultra-15 device. The expression and purity of the proteins were examined by SDS-PAGE. The purified proteins have an additional methionine at the N-terminus and a hexa-histidine tag at the C-terminus.

### 2.3. Analytical Size-Exclusion Column Chromatography

Analytical size-exclusion chromatography of purified MotS_68–242_ and MotS_78–242_ was performed with a Superdex 75 10/300 GL column in buffer A with 300 mM NaCl or 300 mM KCl at a flow rate of 0.6 mL min^−1^. Ovalbumin (43 kDa) and IgG (158 kDa) were used as size markers (Cytiva, Tokyo, Japan).

### 2.4. Crystallization

Crystal screening was performed by the sitting-drop vapor-diffusion technique with the commercially available screening kits Wizard Classic I and II, Wizard Cryo I and II (Rigaku, Tokyo, Japan), and Crystal Screen I and II (Hampton Research, Aliso Viejo, CA, USA) at 277 K or 293 K. Each drop was prepared by mixing 0.5 µL of protein solution (5–10 mg mL^−1^) with 0.5 µL of reservoir solution and equilibrating to 70 µL of reservoir solution. After initial screening, the crystallization conditions were optimized by varying the precipitant concentration, pH, and additives using the sitting-drop vapor-diffusion method.

Crystals of MotS_68–242_ purified in 150 mM NaCl were grown from the drop prepared by mixing 0.5 µL of 6 mg mL^−1^ protein solution with 0.5 µL of reservoir solution containing 1.26 M ammonium sulfate and 0.1 M HEPES-NaOH pH 7.5 at 293 K. The space group of the crystals was orthorhombic *C*2 with unit cell dimensions of a = 83.0, b = 80.7, c = 55.2 Å, and β = 100.4°. Crystals were soaked in a cryo-protectant solution containing 10% (*v*/*v*) glycerol and 90% (*v*/*v*) of the reservoir solution and frozen in liquid nitrogen. Crystals of MotS_68–242_ purified in 300 mM NaCl were grown from the drop prepared by mixing 0.5 µL of 10 mg mL^−1^ protein solution with 0.5 µL of reservoir solution containing 20% PEG-8000 and 0.1 M HEPES-NaOH pH 7.5 at 293 K. The space group of the crystals was orthorhombic *C*2 with unit cell dimensions a = 82.5, b = 79.4, c = 55.9 Å, and β = 99.4°. Crystals were soaked in a solution containing 10% (*v*/*v*) glycerol, 20% PEG-8000, 0.05 M HEPES-NaOH pH 7.5, and 300 mM NaCl and frozen in liquid nitrogen. Crystals of MotS_68–242_ purified in 300 mM KCl were grown from the drop prepared by mixing 0.5 µL of 11 mg mL^−1^ protein solution with 0.5 µL of reservoir solution containing 40% PEG-300 and 0.1 M Na_2_HPO_4_/citric acid pH 4.2 at 293 K. The space group of the crystals was orthorhombic *C*2 with unit cell dimensions a = 82.7, b = 80.0, c = 55.9 Å, and β = 98.8°. Crystals were soaked in a solution containing 40% PEG300, 0.05 M Na_2_HPO_4_/citric acid pH 4.2, and 300 mM KCl and frozen in liquid nitrogen.

### 2.5. Data Collection and Structural Determination

X-ray diffraction data were collected at synchrotron beamlines BL41XU and BL45XU in SPring-8 (Sayo, Japan) with the approval of the Japan Synchrotron Radiation Research Institute (JASRI) (Proposal No. 2018B2569, 2019A/B2551, and 2020A/B2574). Crystals were mounted in nitrogen gas flow at 100 K for X-ray diffraction data collection. The diffraction data were processed with MOSFLM [37] and were scaled with AIMLESS [38]. The statistics of the diffraction data are summarized in Table S2. The initial phase of the MotS_68–242_ crystal was calculated by molecular replacement with the Phenix program [39] using the PomBc5 structure (PDB ID 3wpw) as a search model. The atomic model of MotS_68–242_ was built with Coot [40] and refined to 1.89 Å resolution with Phenix. The refined structure was used as the search model for molecular replacement with Phenix for MotS_68–242_ in 300 mM NaCl and 40 mM NaCl/300 mM KCl. The atomic models of MotS_68–242_ in 300 mM NaCl and in 40 mM NaCl/300 mM KCl were refined to 1.89 Å and 1.90 Å resolution with Phenix, respectively. The structural refinement statistics are summarized in Table S2.

### 2.6. Far-UV CD Spectroscopy

The purified protein samples in buffer A with 300 mM NaCl were diluted with 20 mM Tris-HCl (pH 8.0) solution containing 300 mM NaCl for the high NaCl condition (300 mM NaCl) or with 300 mM KCl for the low NaCl condition (25 mM NaCl/275 mM KCl). We prepared three independent samples for each salt concentration. The protein concentrations of the samples used for the CD measurements are summarized in Table S3.

Far-UV CD spectra (200–250 nm) were collected on a Jasco-720W spectropolarimeter (JASCO Co., Tokyo, Japan) using square quartz cells with a 1 mm path length. Spectra were obtained by averaging four successive accumulations with a wavelength step of 0.5 nm at a rate of 20 nm/min, response time of 8 s, and bandwidth of 2.0 nm. Measurements were repeated three times for each sample at 303 K, and the obtained spectra were averaged. The secondary structure contents were estimated by K2D3 [41] using the CD data.

## 3. Results

### 3.1. Structure of Bs-MotS_68–242_

**Figure 2:**
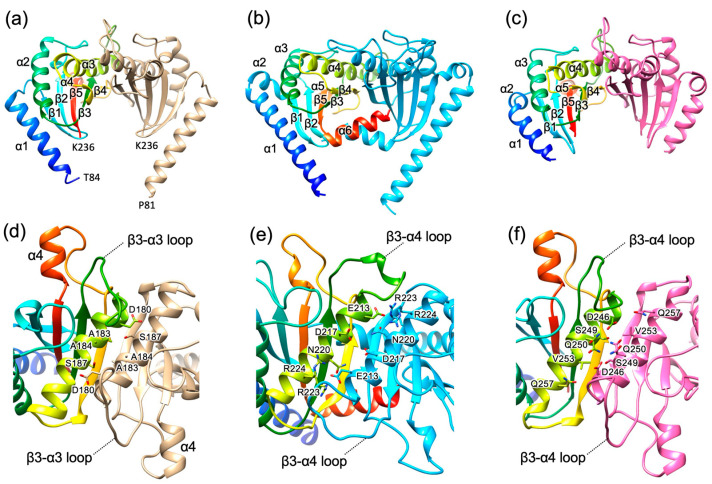
Structural comparison of the periplasmic region of the stator B-subunit from *B. subtilis* (Bs-MotSc), *Salmonella* MotBc (St-MotBc), and *V. alginolyticus* PomBc (Va-PomBc). (**a**) Cα ribbon representation of Bs-MotSc dimer structure in 92 mM NaCl. One subunit is colored in a rainbow from blue at the N-terminus to red at the C-terminus, and the other is shown in light brown. (**b**) Structure of *Salmonella* MotBc (St-MotBc) dimer (PDB ID 2ZVY). One subunit is colored in rainbow, and the other is in cyan. (**c**) Structure of *V. alginolyticus* PomBc (Va-PomBc) dimer (PDB ID 3WPW). One subunit is colored in rainbow, and the other is in pink. (**d**–**f**) Close-up top view of the dimer interface in (**a**–**c**). The side-chains of residues that contribute to inter-subunit interactions are shown as stick models with labels.

We expressed His-tagged Bs-MotS_68–242_ in *E. coli* and purified by Ni-affinity chromatography followed by size exclusion chromatography (SEC). The crystals suitable for X-ray analysis were grown from a solution containing 92 mM NaCl. The crystal structure of Bs-MotS_68–242_ was determined at 1.89 Å resolution (Figure 2a). The asymmetric unit of the crystal contains a Bs-MotS_68–242_ dimer (Sub-A and Sub-B) related by a pseudo-two-fold symmetry. The N-terminal 15 residues of Sub-A, 12 residues of Sub-B, and C-terminal 6 residues of both subunits were not modeled because of poor electron density.

The overall structure of Bs-MotS_68–242_ resembles those of St-MotBc [20] and Va-PomBc [33] (Figure 2 and Figure S1). Bs-MotS_68–242_ consists of an N-terminal α-helix (α1) and a single core domain composed of three α-helices (α2, α3, and α4) and five β-strands (β1, β2, β3, β4, and β5), which includes an OmpA-like domain. α1 of Bs-MotS_68–242_ projected from the core domain as in St-MotBc. A short helix following α1 of St-MotBc and Va-PomBc does not exist in Bs-MotS_68–242_, and α1 of Bs-MotS_68–242_ is connected to β1 by a loop composed of five residues. The conformation of the loop connecting β3 and α3 (the β3–α3 loop) of Bs-MotS_68–242_ differs from the corresponding loop (the β3–α4 loop) in St-MotBc and Va-PomBc. The β3–α4 loop includes a putative PGB site and is a highly flexible loop [20,33]. Therefore, the conformational difference in the loop reflects the flexibility of the loop. A short helix (α4) exists between β4 and β5 as in Va-PomBc and MotBc from *Helicobacter pylori* (Hp-MotB_C_) [42]. The C-terminal region following β5 is disordered. This region is also disordered in Va-PomBc but forms an α-helix in St-MotBc and Hp-MotBc [20,42].

The subunit arrangement of the Bs-MotS_68–242_ dimer resembles those of the St-MotBc and the Va-PomBc dimers (Figure 2). The subunit interface of Bs-MotSc is formed by α3 and β4. β4 forms an inter-subunit hydrogen bond with β4 of the other subunit, forming a ten-stranded inter-subunit β-sheet, and the side chains of α3 interact with those of α3 in the other subunit. These structural features are conserved in St-MotBc and Va-PomBc. However, the helix–helix interaction of Bs-MotS_68–242_ is hydrophobic, whereas those of St-MotBc and Va-MotBc are hydrophilic.

### 3.2. Bs-MotSc Forms a Stable Dimer Regardless of Sodium Concentration

**Figure 3:**
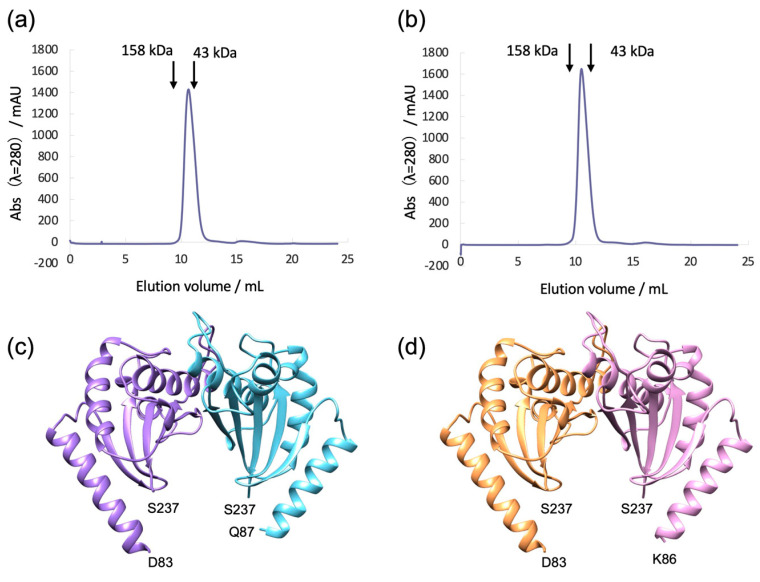
SEC profiles and structures of Bs-MotSc at high and low sodium concentrations. (**a**,**b**) Analytical SEC profiles of purified MotS_68–242_ in 300 mM NaCl (**a**) and 300 mM KCl (**b**). The arrows indicate elution volumes of Ovalbumin (43 kDa) and IgG (158 kDa). (**c**,**d**) Structure of the Bs-MotSc dimer in 300 mM NaCl (**c**) and 40 mM NaCl/300 mM KCl (**d**).

To examine the Na^+^-dependent folding and dimerization of Bs-MotSc, we performed SEC analysis of Bs-MotS_68–242_ in 300 mM NaCl or 300 mM KCl at pH 8.0 (20 mM Tris-HCl). Bs-MotS_68–242_ eluted as a single peak under the conditions, and both elution profiles were almost identical to each other (Figure 3a,b). This result suggests that the Na^+^-dependent structural change in Bs-MotSc is local and small. The apparent molecular mass estimated from the peak position is close to the molecular mass of the Bs-MotS_68–242_ dimer (41 kDa). Therefore, Bs-MotS_68–242_ is in a folded state and forms a dimer even in the absence of Na^+^.

### 3.3. Structure of Bs-MotS_68–242_ at High and Low Na^+^ Concentrations

To investigate the Na^+^-dependent structural change and Na^+^-binding site, we determined the crystal structures of Bs-MotS_68–242_ at high and low Na^+^ concentrations. We purified Bs-MotS_68–242_ at 300 mM NaCl or 300 mM KCl and crystallized them by the sitting-drop vapor-diffusion method. The initial crystallization drops of Bs-MotS_68–242_ purified at 300 mM NaCl and 300 mM KCl contained 170 mM NaCl and 40 mM NaCl/150 mM KCl, respectively. The crystals were soaked in a solution containing 300 mM NaCl or 40 mM NaCl/300 mM KCl before X-ray data collection. The crystal structures of Bs-MotS_68–242_ at 300 mM NaCl and 40 mM NaCl/300 mM KCl were determined at 1.89 Å and 1.9 Å resolutions, respectively (Figure 3c,d). Both form homodimers. The N-terminal 15 to 19 residues and C-terminal 5 residues were disordered. We superimposed both structures and found no significant difference between them. The root mean square deviation of the corresponding Cα atoms is 0.182 Å. To find the Na^+^-binding site, we calculated the difference Fourier map using the diffraction data at 300 mM NaCl and KCl, because the cell parameters of both crystals are quite similar. No significant density appeared, suggesting that Na^+^ may bind to the N-terminal disordered region of Bs-MotS_68–242_.

### 3.4. Na^+^-Dependent Coil–Helix Transition in the N-Terminal Disordered Region

**Figure 4:**
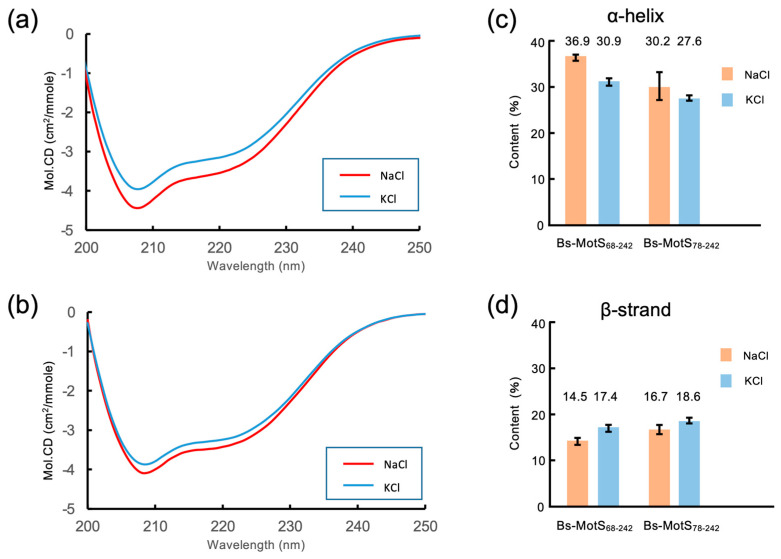
Far-UV CD spectra of Bs-MotSc in high and low sodium concentrations. (**a**,**b**) CD spectra of Bs-MotS_68–242_ (**a**) and Bs-MotS_78–242_ (**b**) at 300 mM NaCl (NaCl) and 25 mM NaCl/275 mM KCl (KCl). (**c**,**d**) Estimated secondary structure contents from the CD data.

To detect the Na^+^-dependent conformational change in Bs-MotSc, we measured the far-UV CD spectra of Bs-MotS_68–242_ at 300 mM NaCl and 25 mM NaCl/275 mM KCl (Figure 4). The CD spectra show a significant difference, indicating that Na^+^ induces a secondary structure change in Bs-MotS_68–242_. We calculated the secondary structure content using these data and found that the α-helix content at 300 mM NaCl is about 6% higher than that at 25 mM NaCl/275 mM KCl, which corresponds to three turns of α-helix (10.5 residues). In contrast, the difference in the β-strand content is almost within the margin of error. These results, together with the crystal structures, suggest that the Na^+^-dependent conformational change is a coil–helix transition in the N-terminal disordered region of Bs-MotS_68–242_.

To confirm this idea, we prepared Bs-MotS_78–242_ and measured the CD spectra at high and low sodium concentrations (Figure 4). The CD spectrum at 300 mM NaCl was almost the same as that at 25 mM NaCl/275 mM KCl. The secondary structure content of Bs-MotS_78–242_ was not significantly changed by the Na^+^ concentration. Thus, we conclude that the region between residues 68 and 77 forms a short helix at a high Na^+^ concentration but not at a low Na^+^ concentration.

### 3.5. Possible Na+-Binding Sites in Bs-MotSc

**Figure 5:**
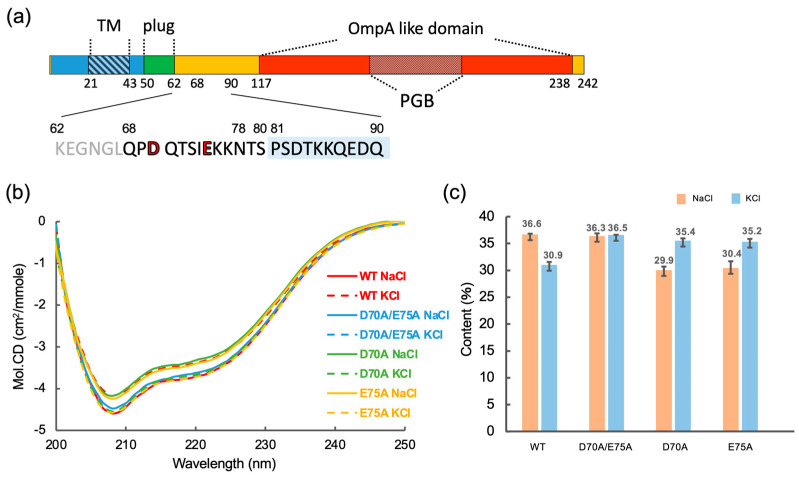
Far-UV CD spectra of Bs-MotSc mutant variants. (**a**) Primary structure of Bs-MotS. The N-terminus of the α1 helix in the Bs-MotSc structure is shaded in light blue. The mutation sites are highlighted in red font. (**b**) CD spectra of Bs-MotS_68–242_ without mutation (wild-type) and with mutations (D70A, E75A, and D70A/E75A) at 300 mM NaCl (NaCl) and 25 mM NaCl/275 mM KCl (KCl). (**c**) The helical content was estimated from the CD data.

The N-terminal disordered region of Bs-MotS_68–242_ contains two acidic residues, D70 and E75, that are likely to interact with Na^+^. Therefore, we constructed three mutant variants, D70A, E75A, and D70A/E75A of Bs-MotS_68–242_, and measured their far-UV CD spectra at high (300 mM NaCl) and low (25 mM NaCl/275 mM KCl) Na^+^ concentrations (Figure 5). All mutant proteins purified as a dimer as judged from the peak position of their SEC profiles (Figure S2). The CD spectrum of D70A/E75A shows the same profile regardless of the Na^+^ concentration and is identical to that of wild-type Bs-MotS_68–242_ at a high sodium concentration. The α-helix content of D70A/E75A is almost at the same level as that of wild-type Bs-MotS_68–242_ at a high sodium concentration, suggesting that the double mutation induces helix formation in the N-terminal disordered region. In contrast, the CD spectra of Bs-MotS_68–242_ with either D70A or E75A show a difference between high and low sodium concentrations with the opposite behavior to the wild-type Bs-MotS_68–242_. Their α-helical content was high at a low Na^+^ concentration and low at a high Na^+^ concentration. These results suggest that D70 and E75 are key residues for the Na^+^-dependent coil–helix transition of the N-terminal region of Bs-MotS_68–242_ and that they co-operatively contribute to the conformational change in this region.

## 4. Discussion

In this study, we determined the structures of Bs-MotS_68–242_, which is the first structure available of the periplasm domain of the stator B-subunit from gram-positive bacteria. The PG layer of gram-positive bacteria differs from that of gram-negative bacteria [43] in thickness, structure, and chemical constituents. Therefore, the stator B-subunit of gram-positive bacteria is expected to have some features distinct from those of gram-negative bacteria. However, the overall structure of the core domain resembles that of gram-negative bacteria (Figure 2), suggesting that the stator B-subunits recognize the PG layer that is common to both gram-positive and gram-negative bacteria.

The stator unit diffuses in the cell membrane in an inactive state. When it contacts the rotor, it tightly binds to the PG layer through the B-subunit dimer for torque generation [20,21,33]. Since the periplasmic domain of the B-subunit dimer is too short to reach the PG layer, a large conformational change is needed in the B-subunit. St-MotB and Va-PomB rearrange the N-terminal helix of their periplasmic domain to an extended conformation for PG binding [20,21,33]. The structure of Bs-MotS_68–242_ resembles St-MotBc and Va-PomBc, suggesting that Bs-MotS adopts structural rearrangement in the N-terminal helix of its periplasmic domain as in St-MotB and Va-PomB.

Bs-MotPS associates with and dissociates from the rotor depending on the external Na^+^ concentration [34]. Previous HS-AFM observations of the purified Bs-MotPS complex indicated that Bs-MotSc appeared clearly at 150 mM NaCl in the AFM movie images but disappeared at 100 mM NaCl [36]. Based on these observations, a Na^+^-dependent folding model has been proposed in which the periplasmic domain of MotS is unfolded at low NaCl concentrations and folds into a dimeric structure at high NaCl concentrations, and through this process, Bs-MotPS associates with and dissociates from the rotor. However, our crystal structures and SEC analyses contradict this model. The crystal structures of BsMotS_68–242_ at 40 mM NaCl/300 mM KCl, 92 mM NaCl, and 300 mM NaCl showed almost an identical dimer structure (Figure 2a and Figure 3c,d), indicating that no unfolding occurs at less than 100 mM NaCl. SEC experiments at 300 mM NaCl and 300 mM KCl also showed that Bs-MotS_68–242_ forms a dimer at a low NaCl concentration in solution (Figure 3a,b). These results indicate that Bs-MotSc forms a homodimer and that no unfolding occurs even at 0 mM NaCl. So, why does Bs-MotSc disappear at low NaCl concentrations in the HS-AFM observation? One possible reason is that the periplasmic domain is highly mobile at a low NaCl concentration, whereas it is fixed at a high NaCl concentration.

The far-UV CD measurements demonstrated that the coil–helix transition occurs in a region containing residues 68 to 77 and depends on the Na^+^ concentration (Figure 4). The Na^+^-dependent increase in the α-helical content estimated from the CD data is about 10 residues, which is consistent with the number of residues in this region. This region in the D70A/E75A protein was always helical, supporting the Na^+^-dependent coil–helix transition of the region (Figure 4).

**Figure 6:**
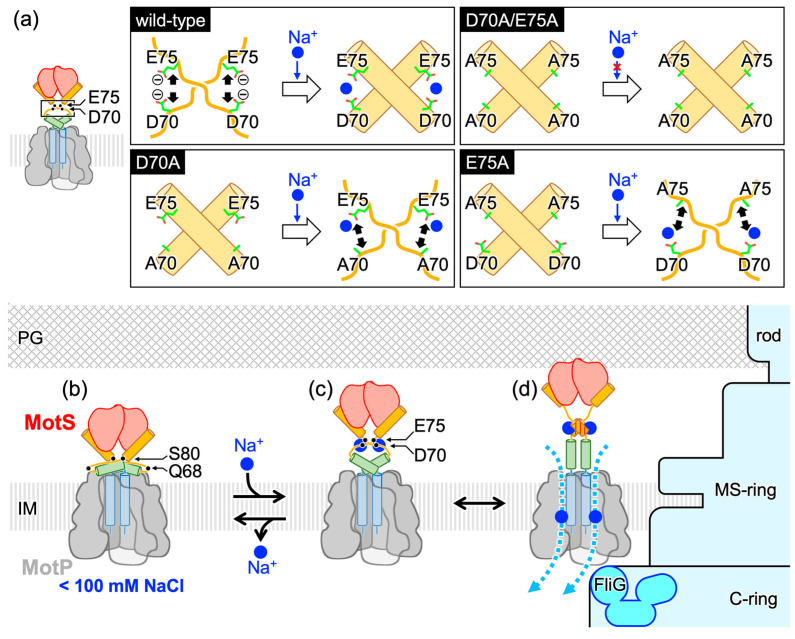
A model for the Na^+^-dependent assembly of the Na^+^-driven Bs-MotPS stator. (**a**) A model for the Na^+^-dependent coil–helix transition of residues 68 to 77 in Bs-MotS. At a low sodium concentration, D70 in one subunit and E75 in the other in the Bs-MotS dimer repel each other by their negative charge, preventing the α-helix formation in the residues 68–77 region (wild-type, left). At a high sodium concentration, the negative charges are neutralized by the binding of Na^+^ ions, and the α-helices are formed (wild-type, right). Since the D70A/E75A mutant protein has no negative charge, residues 68 to 77 from an α-helix (D70A/E75A). In the D70A mutant protein, the Na^+^ binding to E75 of one subunit destabilizes the α-helix formation in the other subunit probably due to repulsion between the bound Na^+^ and A70 (D70A). Similarly, the E75A mutant protein cannot form the α-helix in the residues 68–77 region (E75A). Black arrows indicate the repulsive force. (**b**,**c**) A model for the assembly of the Bs-MotPS stator. (**b**) At low sodium concentrations, the stator adopts a compact form and diffuses in the cell membrane. (**c**) At high sodium concentrations, two Na^+^ ions bind to the interface between D70 of one subunit and E75 of the other subunit. The binding of Na^+^ ions induces the coil-to-helix transition in the disordered region (residues 68 to 80). (**d**) When MotPS comes in contact with the C-ring, the N-terminal helix of Bs-MotSc changes its conformation to bind to the PG layer, as in St-MotB and Va-PomB.

D70 and E75 contribute in a complex way to the coil–helix transition (Figure 5). The α-helical content of the D70A, E75A, and D70A/E75A variants of Bs-MotS_68–242_ at a low Na^+^ concentration was at the same level as in the wild-type fragment at a high Na^+^ concentration, demonstrating that the D70A and E75A mutations stabilize the α-helix structure. However, the α-helical contents of the D70A and E75A variants decreased at a high Na^+^ concentration, whereas that of the D70A/E75A variant at a high Na^+^ concentration had the same level of α-helical content as the wild-type protein. This behavior suggests that D70 and E75 cooperatively contribute to the coil–helix transition. If residues 68 to 77 form an α-helix, D70 and E75 will be in opposite positions on the helix and cannot interact with each other. Thus, D70 of one subunit may interact with E75 of the other subunit in the dimer, and Na^+^ may affect the inter-subunit interactions. The binding of Na^+^ to D70 (E75) of one subunit of the E75A (D70A) mutant protein may destabilize the formation of the α-helix of the other subunit (Figure 6a).

Based on these studies, we propose a new model for the Na^+^-dependent conformational change in Bs-MotS (Figure 6). When the external Na^+^ concentration is low, Bs-MotSc exists as a compact dimer. However, the N-terminal connection of the OmpA-like domain to the transmembrane and plug regions is flexible because residues 68 to 80 are disordered (Figure 6b). When the external Na^+^ concentration increases enough, D70 of one subunit and E75 of the other subunit may cooperatively bind Na^+^, inducing the disordered region to form an α-helix. This conformational change rigidifies the chain connecting the plug and the periplasm domain, and the orientation of the OmpA-like domain is positioned to be ready for binding to the PG layer (Figure 6c). When MotP contacts with FliG in the C-ring, a structural rearrangement occurs in the N-terminal helix of Bs-MotSc, as in St-MotB and Va-PomB, to bind to the PG layer (Figure 6d).

The Na^+^-dependent coil–helix transition may prevent the incorporation of the stator units into the motor under low sodium concentrations. Our study suggests that D70 and E75 are key residues for the Na^+^-dependent behavior of the BS flagellar motor. Experiments to test the motility and growth at low and high NaCl concentrations of cells lacking *motAB* and *motPS* that produce the D70A, E75A, and D70A/E75A variants of MotS with MotP are clearly suggested for future work.

Va-PomAB also shows Na^+^-dependent association/dissociation [30]. However, the amino acid sequence between the plug region and the N-terminal helix of PomBc differs from that of Bs-MotS (Figure S1). Since Va-PomAB targets the T-ring, which does not exist in the flagellum of *B. subtilis*, the Na^+^-dependent association/dissociation of Va-PomAB may be controlled by a different mechanism than Bs-MotPS.

## 5. Conclusions

The Na^+^-dependent association/dissociation of Bs-MotPS is caused by the conformational change of the periplasmic region of Bs-MotS. The crystal structures and SEC analyses of MotS_68–242_ at high and low Na^+^ concentrations revealed that the periplasmic region of Bs-MotS is in a folded state and forms a homodimer regardless of the external Na^+^ concentration. The far-UV CD measurements indicated that the Na^+^-dependent coil-helix transition occurs in a region containing residues 68 to 77 and that D70 and E75 are responsible for the structural transition. We propose that the Na^+^-dependent coil–helix transition triggers the Na^+^-dependent association/dissociation of Bs-MotPS.

## Data Availability

The atomic coordinates have been deposited in the Protein Data Bank, https://www.wwpdb.org under accession codes 9LJK (BsMotS_68–242_ in 92 mM NaCl), https://www.rcsb.org/structure/9LJK (accessed on 20 February 2025), 9LJL (BsMotS_68–242_ in 300 mM NaCl), https://www.rcsb.org/structure/9LJL (accessed on 20 February 2025), and 9LJM (BsMotS_68–242_ in 40 mM NaCl/300 mM KCl), https://www.rcsb.org/structure/9LJM (accessed on 20 February 2025).

## Supplementary Materials

The following supporting information can be downloaded at: https://www.mdpi.com/article/10.3390/biom15020302/s1, Figure S1: Structure-based amino acid sequence alignment of Bs-MotS (BsMS), St-MotB (StMB), and Va-PomB (VaPB); Figure S2: The SEC elution profiles of Bs-MotS_68–242_ with or without mutations at 300 mM NaCl; Table S1: Strains and plasmids used in this study [44]; Table S2: Summary of X-ray data collection and refinement statistics; Table S3: Summary of protein concentrations (mg/mL) of the samples used for the CD measurements.
