# Supplementary material for: Sodium-Dependent Conformational Change in Flagellar Stator Protein MotS from Bacillus subtilis
Source: Biomolecules. 2025 Feb 18;15(2):302. doi: 10.3390/biom15020302 (PMC11853200; doi:10.3390/biom15020302)
Supplement: Supplementary file 1 [file biomolecules-15-00302-s001.zip › biomolecules-3480333-supplementary.pdf]

```
                                                  TM                   Plug
BsMS    1 ---MKLRRER FERRNGSGKN SQSSSSWMVT FTDLITLILV FFILLFSMSQ IDLQKFKAAV  57
StMB    1 MKNQAHPIVV VKRRRHKPHG GGAHGSWKIA YADFMTAMMA FFLVMWLISI SSPKELIQIA  60
VaPB    1 ---------M DDEDNKCDCP PPGLPLWMGT FADLMSLLMC FFVLLLSFSE MDVLKFKQIA  51

           Plug
BsMS   58 DSIQKEGNGL ---------- ---------- ---------- ---------- ----------  67
StMB   61 EYFRTPLAT- AVTGGNRIAN SES------- ---------- ---------- ----------  82
VaPB   52 GSMKFAFGVQ NQLEVKDIPK GTSIIAQEFR PGRPEPTPID VIMQQTMDIT QQTLEFHEGE 111

                                                                 α1
BsMS   68 ---------- ---------- ---------- QPDQTSIEKK -NTSPSDTKK QEDQQDQLLK  96
StMB   83 ---------- ---------- -----PIPGG GDDYTQQQGE VEKQPNIDEL KKRMEQSRLN 117
VaPB  112 SDRAGGTKRD EGKLTGGQSP ATSTQNNESA EADMQQQQS- ---------- --KEMSQEME 158
                                                                         α1

             α1                      β1       β2                       α2
BsMS   97 KVNTYIKDN- ----HLK--- AQMTAKRDER GVVLVLQE-- AV-LFDTGEA KVLKNAETLL 145
StMB  118 KLRGDLDQLI ESDPKLRALR PHLKIDLVQE GLRIQIIDSQ NRPMFKTGSA EVEPYMRDIL 177
VaPB  159 TLMESIKKAL --EREIEQ-- GAIEVENLGQ QIVIRMRE-- -KGAFPEGSA FLQPKFRPLV 211
             α1          α2          β1       β2                       α3

              α2            β3                           α3
BsMS  146 HQIAVLLQTI --PNDIQVEG HTDSRNI-ST YRYPSNWELS AARASGVIQY FTSKEKLPSK 202
StMB  178 RAIAPVLNGI --PNRISLAG HTDDFPYANG EKGYSNWELS ADRANASRRE LVAGGLDNGK 235
VaPB  212 RQIAELVKDV --PGIVRVSG HTDNRPL-DS ELYRSNWDLS SQRAVSVAQE MEKVRGFSHQ 268
              α3            β3                           α4

             β4                α4       β5
BsMS  203 RFIAVGYADT KPVKDNKTNE HMKENRRVEI VIKKSKTTSS ---------- ---------- 242
StMB  236 VLRVVGMAAT MRLSDRGPDD --AINRRISL LVLNKQAEQA ILHENAESQN EPVSVLQQPA 293
VaPB  269 RLRVRGMADT EPLLPNDSDD NRALNRRVEI SIMQGEPLYS EEVPVIQ--- ---------- 315
             β4                α5       β5        α6

StMB  294 AAPPASVPTS PKAEPR_309
```

Figure S1. Structure-based amino acid sequence alignment of Bs-MotS (BsMS), St-MotB (StMB), and Va-PomB (VaPB). The regions whose structures have been determined are highlighted by bold characters. The secondary structural elements are labeled above (for Bs-MotS) and below (for St-MotB and Va-PomB) the sequence. The residues that form the a-helix and b-strand are colored by red and blue, respectively. The transmembrane region (TM) and the plug region (Plug) are indicated by cyan and green boxes, respectively.

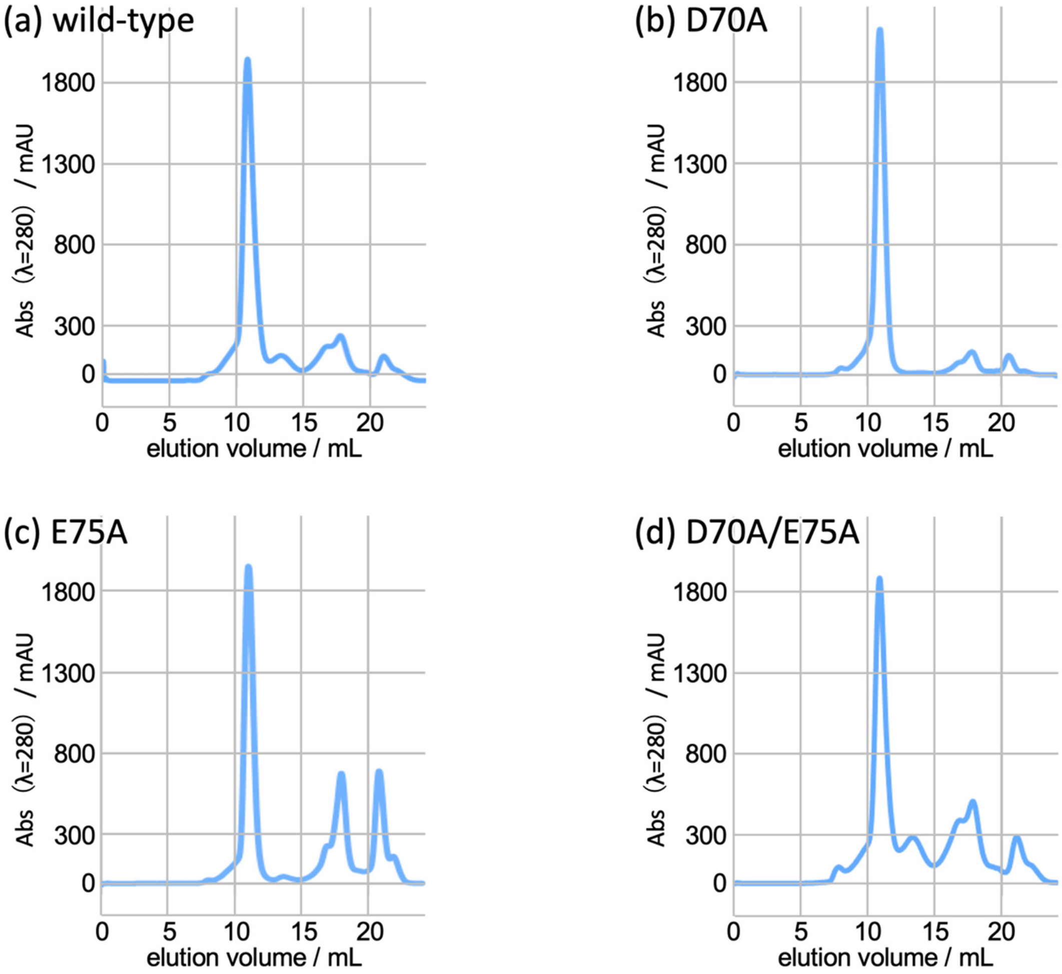

Figure S2. The SEC elution profiles of Bs-MotS$_{68\text{-}242}$ with or without mutations at 300 mM NaCl. The elution volume of the peak indicates that all of the Bs-MotS$_{68\text{-}242}$ variants form a dimer.

Table S1. Strains and plasmids used in this study.

Strain or plasmid	Description	Source or reference
<u>*E. coli* strains</u>		
DH5α	F^- λ^- *recA1 hsdR17 endA1 supE44 thi-1 relA1 gyrA96*Δ(*argF-lacZYA*) U169 φ80d*lacZ*ΔM15)<br>(Recipient for cloning experiments)	[44]
BL21(DE3)	F^- *ompT hsdS*(r_B^- m_B^-) *gal dcm* λ(DE3)<br>(Host for overproduction of proteins)	Novagen
<u>Plasmids</u>		
pET21b	Expression vector (ampicillin resistant, T7 promoter)	Novagen
$MotS_{68\text{-}242}$ expression plasmid	pET21b/ Bs-$MotS_{68\text{-}242}$-His_6	Naoya Terahara
pMU01	pET21b/ Bs-$MotS_{68\text{-}242}$(D70A)-His6	This study
pMU02	pET21b/ Bs-$MotS_{68\text{-}242}$(E75A)-His6	This study
pMU03	pET21b/ Bs-$MotS_{68\text{-}242}$(D70A/E75A)-His6	This study
$MotS_{78\text{-}242}$ expression plasmid	pET21b/ Bs-$MotS_{78\text{-}242}$-His_6	Naoya Terahara

Table S2. Summary of X-ray data collection and refinement statistics.

	Bs-MotS$_{68\text{-}242}$ at 92 mM NaCl	Bs-MotS$_{68\text{-}242}$ at 300 mM NaCl	Bs-MotS$_{68\text{-}242}$ at 40 mM NaCl/300 mM KCl
**Data collection**			
Space group	*C*2	*C*2	*C*2
Cell dimensions			
a, *b*, *c* (Å)	82.99, 80.73, 55.24	82.51, 79.37, 55.93	82.68, 80.00, 55.88
α, β, γ (°)	90.00, 100.44, 90.00	90.00, 99.42, 90.00	90.00, 98.80, 90.00
Resolution (Å)	54.3-1.89 (1.93-1.89) *	55.2-1.89 (1.93-1.89)	55.2-1.90 (1.94-1.90)
CC1/2	0.993 (0.655)	0.993 (0.731)	0.995 (0.836)
I / σI	8.7 (2.3)	7.5 (2.3)	9.7 (1.8)
Completeness (%)	99.3 (99.8)	99.3 (98.4)	99.5 (97.6)
Redundancy	3.4 (3.4)	3.0 (3.0)	3.5 (3.5)
**Refinement**			
Resolution (Å)	54.3-1.89 (1.96-1.89)	55.2-1.89 (1.94-1.89)	55.2-1.90 (1.95-1.90)
No. reflections	28670 (2854)	27998 (2015)	27769 (1963)
R_{work}/R_{free}	0.175/0.217 (0.290/0.358)	0.179/0.221 (0.256/0.305)	0.211/0.236 (0.326/0.317)
No. atoms			
Protein	2511	2486	2513
Ligand/ion	5	0	0
Water	218	384	170
B-factors			
Protein	28.0	20.9	38.3
Ligand/ion	58.6	-	-
Water	36.0	29.7	41.3
Ramachandran plot (%)			
Favored	98.03	96.03	97.03
Allowed	1.97	3.97	2.97
Outliers	0	0	0
R.m.s. deviations			
Bond lengths (Å)	0.007	0.004	0.003
Bond angles (°)	0.865	0.678	0.572

Values in parentheses are for the highest-resolution shell.

Table S3. Summary of protein concentrations (mg/mL) of the samples used for the CD measurements.

	NaCl-1	NaCl-2	NaCl-3	KCl-1	KCl-2	KCl-3
Bs-MotS$_{68\text{-}242}$	0.167	0.167	0.166	0.167	0.167	0.167
Bs-MotS$_{78\text{-}242}$	0.175	0.168	0.166	0.252	0.266	0.218
Bs-MotS$_{68\text{-}242}$ D70A	0.167	0.167	0.169	0.167	0.167	0.167
Bs-MotS$_{68\text{-}242}$ E75A	0.178	0.176	0.176	0.171	0.170	0.175
Bs-MotS$_{68\text{-}242}$ D70A/E75A	0.160	0.160	0.160	0.167	0.167	0.168

NaCl-1, NaCl-2, and NaCl-3: 300 mM NaCl

KCl-1, KCl-2, and KCl-3: 25 mM NaCl/275 mM KCl
